# Multidisciplinary Telemedicine Care for Tourette Syndrome: Minireview

**DOI:** 10.3389/fneur.2020.573576

**Published:** 2020-12-18

**Authors:** Shan-shan Cen, Jun Yu, Qiao Wang, Wissam Deeb, Kai-liang Wang, Aparna Wagle Shukla, Irene Malaty, Adolfo Ramirez-Zamora, Jian-guo Zhang, Wei Hu, Fan-gang Meng

**Affiliations:** ^1^Program in Movement Disorders and Neurorestoration, Department of Neurology, Fixel Institution for Neurological Diseases, University of Florida, Gainesville, FL, United States; ^2^Department of Neurology, Xuanwu Hospital of Capital Medical University, Beijing, China; ^3^Department of Functional Neurosurgery, Beijing Neurosurgical Institute, Beijing Tiantan Hospital, Capital Medical University, Beijing, China; ^4^Department of Neurology, University of Massachusetts, Worcester, MA, United States; ^5^Chinese Institute for Brain Research, Beijing, China

**Keywords:** Tourette syndrome, telemedicine, movement disorder, CBIT, tics

## Abstract

Tourette syndrome (TS) is a childhood-onset, chronic neuropsychiatric disorder characterized by multiple motor and vocal tics. TS poses a considerable burden on both patients and health care providers, leading to a major detriment of educational success, occupation, and interpersonal relationships. A multidisciplinary, specialist-driven management approach is required due to the complexity of TS. However, access to such specialty care is often dramatically limited by the patients' locations and the specialists' geographic clustering in large urban centers. Telemedicine uses electronic information and communication technology to provide and support health care when distance separates participants. Therefore, we conducted this mini-review to describe the latest information on telemedicine in the assessment and management of TS and discuss the potential contributions to care for TS patients with a multidisciplinary approach. We believe that telemedicine could be a revolutionary method in improving medical access to patients with TS.

## Introduction

Tourette syndrome (TS) is a complex neuropsychiatric movement disorder characterized by multiple motor tics and at least one vocal/phonic tic lasting for more than 1 year. TS is common with a prevalence of 3–8 per 1,000 children, affecting males more often than females by a ratio of about 4:1 (1). It commonly manifests during childhood but affects children, adolescents, and adults worldwide. Although some tics may be mild, others can result in significant psychosocial, physical, and functional difficulties that impair social activities, academic achievements, and employment performance. Additionally, up to 90% of patients with TS exhibit one or more comorbidities, including attention deficit hyperactivity disorder (ADHD), obsessive–compulsive disorder (OCD), sleep disorders, and other behavioral and psychosocial problems (2). These comorbid conditions cause many patients with TS to suffer from varying degrees of additional functional impairment across multiple domains.

Management of TS requires a combination of therapies to treat tics and comorbid conditions. Patients with mild and non-disabling tics should receive education, counseling, and supportive care. Therapeutic strategies for impairing tics are individually tailored, including behavioral therapy—especially the Comprehensive Behavioral Intervention for Tics (CBIT)—as the first-line option, followed by various standard and emerging pharmacologic treatments, and finally, deep brain stimulation (DBS) surgery (3). Although appropriate treatment may reduce tic frequency and severity, a large proportion of patients have a significantly impaired quality of life (QoL) compared to individuals without TS. Tic severity, greater emotional and behavioral difficulties, especially OCD, ADHD, anxiety, and depression, have shown the most significant impact on QoL (4–7).

In the current care model, evaluation and management of TS require frequent travel to tertiary centers and multiple longitudinal in-person visits. Tics are usually intermittent, fluctuating, and often less pronounced when the patient is examined face to face; thus, they can be difficult to assess during regular visits. The care is usually multidisciplinary, consisting of numerous sessions with various providers. Consequently, this increases the burden on patients and caregivers and contributes to the challenges of obtaining and maintaining comprehensive specialized care. In many areas, a severe shortage of needed specialists such as neurologists, psychiatrists, and psychologists further compound the barriers to health care access, equity, and quality (8, 9). A recent survey showed that in the United States, as many as 26% of adult patients have found it challenging to find a physician that is knowledgeable about TS (10). Furthermore, a recent study has suggested that TS also poses a tremendous economic burden on patients, families, and society. The annual TS-specific costs totaled 3,404€ per patient in Germany, with 18% direct costs and 81% indirect costs (11).

Telemedicine, defined by the Institute of Medicine as “the use of electronic and communications technologies to provide and support health care when distance separates the participants,” brings benefits to patients and health care systems by facilitating timesaving and cost-effective access to tertiary care services (12). Because of the challenges mentioned above in the evaluation and management of TS, telemedicine could be an innovative and particularly attractive approach to improving health care and outcomes for patients with TS. This article aims to review the current status of telemedicine in the assessment and management of TS and discuss the potential contributions to care for TS patients with a multidisciplinary approach.

## Evaluation and Diagnosis

### Tics

TS belongs to a spectrum of tic disorders ranging from a provisional form to those associated with general medical conditions. The diagnosis of a tic disorder is based on historical features and observation of the tics either directly or by video recording to assess tic classification and characteristics. Tics are sudden, rapid, recurrent, non-rhythmic motor movements or phonic productions and are classified into the motor and phonic categories, with each subdivided into simple and complex groups. Simple motor tics are characterized by simple muscle movements such as eye blinking, head jerking, and shoulder shrugging. In contrast, complex motor tics comprise complex patterns of activities such as smelling, touching, hitting, jumping, bending, echopraxia (imitating observed movements), and copropraxia (making obscene gestures). Simple phonic tics include various sounds and noises such as grunting, barking, and hooting. Complex phonic tics involve linguistically meaningful vocalizations and expressions, repetition of words/syllables/phrases, echolalia (repeating other people's words), palilalia (repeating one's own words), coprolalia (obscene words or profanity), or other vocal alterations (3). The severity of tics can be evaluated by The Yale Global Tic Severity Scale, a widely used assessment instrument consisting of the Total Tic Score (composed of separate ratings on the number, frequency, intensity, complexity, and interference of motor and vocal tics) and the Tic Impairment Score (based on the impact of the tic disorder on self-esteem, family life, and social acceptance). Additionally, about 90% of adults (13) and 37% of children (14) report a premonitory urge/sensation—vaguely defined as an urge, mounting internal tension, itch, or feeling—in a crescendo fashion just before a motor or phonic tic. The premonitory urge can be a useful clue to differentiate from other repetitive movements, such as stereotypes and psychogenic movement. The Premonitory Urge for Tics Scale characterizes and quantifies premonitory urges (15). For the clinical diagnosis of TS, both the *Diagnostic and Statistical Manual of Mental Disorders, Fifth Edition* (DSM-5) and the Tourette Syndrome Classification Study Group criteria (16) require the presence of both multiple motor tics and one or more phonic tics with onset before age 18 years.

Of note, a patient with a tic disorder typically has a normal neurologic examination. Possible “soft” neurologic findings may include incoordination, synkinesis (involuntary muscular movements accompanying voluntary movements), and motor restlessness—especially in individuals with comorbid ADHD individuals. There is no definitive diagnostic laboratory test. Neuroimaging studies are also typically normal.

### Comorbidities

In addition to a careful review of the medical, social, and family history for tics and tic-related disorders, the evaluation of a patient with a suspected tic disorder should also include an assessment of the common presence and detrimental effect of the coexisting conditions, as 86–90% of individuals with TS have at least one comorbid neuropsychological problem (17). ADHD symptoms are reported in 21–90% of TS patients and usually precede tics onset of tics by 2–3 years (18). A lifetime comorbid diagnosis of OCD is present in about 50% of TS patients, and the obsessive–compulsive behaviors usually emerge several years after the onset of tics during early adolescence, although an earlier age of onset has also been suggested (19). Generalized anxiety disorder is reported in 19–90% of TS patients with increased rates in children and youth (20). The presence of depression has positively correlated with earlier onset and a longer duration of tics in TS (21). There is a higher prevalence of suicidal ideations and attempts in TS (22). Episodic outbursts, rage, difficulty with aggression, and other disruptive behaviors are common and reported in 25–70% of TS populations (17). Self-injurious behavior in TS correlates with impulsivity and impulse control (23). The frequencies of poor self-concept, reduced self-esteem, antisocial activities, oppositional behaviors, schizotypal traits, and personality disorders are also increased in TS (24).

These additional issues add an extra clinical burden and can cause greater functional and social impairments than that induced by the tics. Health-related QoL assessments have shown that the presence of these comorbidities, rather than tic severity, predicts clinical outcomes (5). Thus, the evaluation of TS requires a multidisciplinary team of experts, including neurologists, psychologists, psychiatrists, and behavior therapists.

### Telemedicine

Many chronic neurological conditions, including movement disorders, impair mobility, cognitive function, and driving ability. As these diseases progress, the patients' ability to access care decreases, leaving those with the greatest need for attention receiving the least care. Particularly, the supply of pediatric and adult neurologists with expertise in childhood-onset movement disorders is limited and unevenly distributed. As such, the need for teleneurology for the evaluation and management of TS and other tics disorders is immense. Telemedicine has been shown to improve specialty care access, allowing earlier evaluation and diagnosis of movement disorders and concomitant neurological, psychiatric, and medical comorbidities (25, 26).

In the realm of movement disorders, using a telemedicine approach to detect, characterize, and monitor the motor and non-motor symptoms and, in turn, reaching a diagnosis of the hypokinetic and hyperkinetic conditions has been primarily studied in the context of Parkinson's disease (PD). National randomized controlled trials (RCTs) have confirmed the feasibility of telemedicine house calls evaluating PD patients without worsening clinical outcomes or caregiver burden or increasing the number of emergency room visits or hospitalizations (27, 28). Many studies have highlighted the feasibility, reliability, and safety of telemedicine and mobile technologies, utilizing video (even just short clips), teleconferencing, wearable devices, or other novel technologies, in motor evaluation of tremors, bradykinesia, upper and lower limb functioning, posture, gait, and dyskinesia in PD patients, with satisfactory patient compliance (29–38). Notably, several studies have shown non-inferiority of teleneurology vs. in-person visits to evaluate and assess PD using the Unified Parkinson's Disease Rating Scale (UPDRS) (37, 39). Furthermore, evaluation of voice and speech impairments in PD patients has been shown to be reliable and comparable via telecommunication vs. face-to-face visits (40). Last but not least, the feasibility and concurrent validity of performing movement assessment via video clips or teleconferencing for pediatric populations have also been reported (41, 42).

While studies demonstrating the feasibility, validity, and diagnostic accuracy of TS and other tic disorders via telemedicine are lacking, one can easily speculate that an interdisciplinary telemedicine approach for evaluating and diagnosing tic disorders, particularly TS, has bright prospects. Like PD, TS is also suitable for evaluation and management via telemedicine since TS is clinically diagnosed and most of the physical exam findings are audio-visual (43) that can be reliably collected using telemedicine technologies. Some of the signs and symptoms can be difficult to adequately elicit and capture in a typical clinical setting due to the fluctuations of symptoms and severity. Specifically, tics are typically less pronounced when the patient is being examined face to face, making telemedicine and less obtrusive remote monitoring particularly appealing.

Additionally, both TS and PD patients present with comorbid emotional, behavioral, and other psychosocial problems, requiring a comprehensive patient-centered interdisciplinary care with the active involvement of Neuropsychology and Psychiatry. Feasibility and validity of cognitive screening with the Montreal Cognitive Assessment (MoCA) in patients with movement disorders via telemedicine have been published (33–38). Telepsychiatry has also been found to be a feasible and comparable alternative to face-to-face contact for evaluating the psychiatric comorbidities for patients, including those with movement disorders, with high patient satisfaction and improved access to care (44–47). Specifically, relevant to TS, ADHD has been the most common disorder treated through telepsychiatry and is considered a reasonable alternative to an in-office visit to provide evidence-based care, especially for underserved populations of youth with ADHD (48, 49). It can help facilitate collaborative models of care to empower primary care providers to manage ADHD within their practices while also ensuring those with more severe and complicated illness getting more specialized care when needed. Similarly, a small study found that telepsychiatry resulted in near-perfect interrater agreement on rating scale scores for OCD, and both in-person and telepsychiatry approaches demonstrated excellent diagnostic reliability (50). Finally, multiple studies confirmed the feasibility and diagnostic accuracy for mood disorders via the telepsychiatry approach (51, 52). Thus, telemedicine can lower barriers for access to the scarce but much-needed subspecialty expertise and bring together a comprehensive multidisciplinary care plan, improving the health and functional outcomes and possibly enhancing the QoL of TS patients and their caregivers.

## Management

After a careful initial assessment of the tics, the presence of comorbid issues, and the resulting impairment in each scenario, either in-person or via telemedicine, an effective, comprehensive therapeutic plan for individuals with tic disorders, even though there is no cure *per se*, consists of a systematic tiered approach of education, behavioral therapy, pharmacotherapy, and then more invasive interventions.

### Tele-Psychoeducation

The first step is to educate the patients, their family, and their school and workplace about the diagnosis, potential comorbid conditions, and indications for therapy.

Children with TS usually experience negative perceptions from their peers (53, 54). Such social rejection and isolation lead to bullying and teasing (55), actively impairing their social development (56). It is critical to educate all patients and their parents, peers, and teachers about the natural course and associated comorbidities of this condition. Children with the disorder can be fully prepared with relevant information, promoting strategies to cope with the surrounding environment and retain self-esteem (57). Simultaneously, it can improve mutual relationships and alter misconceptions.

A couple of studies examined the impact of educating peers about TS have shown positive changes in the attitude and behavioral intentions of those peers toward individuals suffering from TS (58–61). Peers that attended those studies received information by watching a video or had been provided a vignette labeling TS developed in conjunction with a team of experts. On the contrary, one study used short educational statements and failed to show a difference; the reason was thought to be insufficient information delivery (54). A similar study for elementary school teachers provided a 2-h education workshop about TS results in a 5% improvement in knowledge (62). Educating teachers is valuable since they play a vital role in the early detection and initial referral of children in need of mental health care. Besides, numerous studies examined the impact of educating folks about ADHD (1). Studies indicated that providing sufficient information to peers and teachers can alter the misconceptions and change the attitude toward a patient's behavior (63–68). Also, improving parental knowledge about ADHD can enhance treatment compliance and increase enrollment (69, 70). Moreover, the positive effects of psychoeducation intervention have been substantially discussed in other psychological disorders related to TS, such as depression and OCD, etc. (71, 72).

However, the ways of delivering education vary. There are training programs, education group sessions, or just a video-taped program, providing a range of information about TS (66, 67, 69). The literature emphasizes the importance of providing sufficient information since simply giving diagnostic labeling to peers is unhelpful (73), raising the question to find the optimal way to present knowledge effectively and conveniently (74). The satisfaction and feasibility of telemedicine-delivered psychoeducation have been demonstrated by a few studies in PTSD veterans (75), hospice caregivers (76), and stem cell transplant survivors (77). Psychoeducation has also been incorporated in remote management of depression (78, 79). Recently, the International Parkinson's Disease and Movement Disorder Society have sponsored a tele-education Parkinson's disease program for health providers in Douala (Cameroon), successfully delivering lectures through synchronous video conferences. Although post-course patient access remained unchanged, medical knowledge was improved dramatically (80). These preliminary studies prove the feasibility of providing education via telecommunication technology and hint toward potential applications in other fields. Thus, tele-psychoeducation, utilizing modern technologies such as real-time videoconference, would be a reliable method to deliver sufficient information about TS and associated comorbidities with precision. Furthermore, it would allow frequent re-evaluation to monitor long-term changes in behaviors.

### Tele-Behavior Therapy

While supportive care is often sufficient for many individuals with milder tics, the presence of psychosocial problems, tic-induced musculoskeletal/physical difficulties, and disruption of school work settings would indicate a need to initiate behavioral or pharmacologic tic-suppressing therapy to reduce tics to a degree where they are no longer causing significant problems.

Habit reversal therapy (HRT), notably CBIT, has become the gold standard and initial intervention for managing and coping with tics (56) without the adverse effects associated with pharmacotherapies, such as cognitive blunting, depression, weight gain, and motor dysfunction (81). CBIT is a well-established therapy consisting of HRT core component—awareness training and competing response training—along with relaxation training and functional intervention (82). Multiple clinical randomized trials and meta-analysis have consistently determined its efficacy (83, 84). Unfortunately, CBIT is not widely available due to a paucity of skilled providers (84–86).

So far, a few studies have investigated the delivery of CBIT remotely, such as the use of a website interface—TicHelper.com (87). A 2012 pilot RCT study has demonstrated comparably significant tic reduction among the 10 TS patients receiving CBIT via videoconference vs. the nine patients receiving face-to-face CBIT (25). Another 2016 RCT examined the delivery of CBIT via the Voice over Internet Protocol (VoIP) approach vs. the waitlist control and found more significant reductions in clinician-rated and patient-reported tic severity among the 12 patients in the VoIP-delivered CBIT group after 10 weeks (88). Additionally, in 2019, internet-delivered behavioral therapies were again rated as highly acceptable, credible, and satisfactory in a small 23-patient pilot study (89). Jakubovski et al. (90) created a new and sophisticated internet-delivered CBIT (iCBIT) program and designed a multicenter and prospective RCT in Germany to discover the efficacy of this program, yet there were no results published so far. Studies regarding telemedicine delivered behavior therapy for tics are summarized in Table 1.

**Table 1:** Summary of studies for the telemedicine delivered behavior therapy for tics.

**Study (author and date)**	**Design**	**Participants**	**Intervention**	**Equipment**	**Assesment points**	**Outcome measure**	**Results**
Himle et al. (91)	Multiple-baseline	Three children with TS, ages of 8–17	Eight, 1-h session of HRT, delivered 1 week apart via VC	Sony HDX8000 VC unit	Pre and post	In-home videotape measured tic frequency, YGTSS, TEI-SF, TAQ, WAI	All three children demonstrated tic reduction compared to baseline (PND = 89%, 100%, 69%). All participants and family reported satisfaction (TEI-SF rating 4–5; TAQ rating 5–7) and working alliance (WAI-SF rating 5–7)
Himle et al. (25)	RCT	20 children ages of 8–17 assigned to CBIT over VC (*n* = 10) or traditional F2F (*n* = 10) delivery	8-session CBIT administered over 10 weeks	Sony PCS- XG80 VC Unit	Pre and post and follow-up (4 months)	YGTSS, CGI- S&CGI-I, PTQ, WAI, TAQ	No between-group difference in change on YGTSS, CGI-I, and PTQ at post-treatment and follow- up; strong acceptability and therapeutic alliance
Ricketts et al. (88)	RCT	20 children ages of 8–16 assigned to CBIT-VoIP (*n* = 12) or waitlist (*n* = 8) group	Two 1.5-hour CBIT sessions followed by six 1-h sessions over 10 weeks	Skype	Pre and post	YGTSS, CGI-I, PTQ, CPTR, CSQ, TAQ, VSQ	Significant reduction on YGTSS, CGI-I, and PTQ in CBIT-VoIP group compared to waitlist. High treatment satisfaction and therapeutic alliance.
Andren et al. (89)	RCT	23 children ages 7–17 assigned to BIP TIC HRT (*n* = 11) or BIP TIC ERP (*n* = 12) group	10 chapters of HRT/ERP delivered over 10 weeks	BIP (Barninternet projektet) (w ww.bup.se/bi p)	Pre and post and follow- up (3, 6, 12 months)	YGTSS, CGI- S&CGI-I, PUTS, GTS-QOL, PTQ	High feasibility and satisfaction. Significant reduction on CGI-I, PTQ, and GTS-QOL in both groups, but no significant reduction on YGTSS for BIP TIC HRT at the primary endpoint (3 months). Therapeutic benefits maintained up to 12 months

*VC, videoconference; F2F, face-to-face; YGTSS, Yale-Global Tic Severity Scale; WAI, Working Alliance Inventory-Short Form; TEI-SF, Treatment Evaluation Inventory-Short Form; TAQ, Treatment Acceptability Questionnaire; CGI-S & CGI-I, Clnician Global Severity & Improvement Scales; PTQ, Parent Tic Questionnaire; VoIP, Voice over Internet Protocol; CPTR, Children's Perception of Therapeutic Relationship; CSQ, Client Satisfaction Questionnaire; VSQ, Videoconferencing Satisfaction Questionnaire; BIP TIC ERP, internet-delivered exposure and response prevention; BIP TIC HRT, internet-delivered habit reversal training; PUTS, Premonitory Urge for Tics Scale; GTS-QOL, Gilles de la Tourette Syndrome-Quality of Life Scale*.

In sum, these studies suggest that tele-CBIT is a promising tool for delivering behavioral therapies for patients with TS/tic disorders and may help increase compliance and adherence by creating alternative avenues for managing tics. These studies do have limitations, however, including small sample sizes and lack of long-term follow-up, making it difficult to draw definitive conclusions on the efficacy of tele-CBIT for the treatment of tics in patients with TS or chronic tic disorders. Data on cost-effectiveness are also lacking.

### Tele-Pharmacotherapy

Pharmacotherapy can be considered when CBIT/HRT alone is insufficient to achieve satisfactory control of the tics and comorbid conditions (1). Recommendations regarding the sequence of pharmacologic therapy vary. In general, guanfacine, clonidine, topiramate, clonazepam, and baclofen are considered tier 1 medications and recommended for milder tics. Atypical and typical antipsychotics (including aripiprazole, risperidone, olanzapine, ziprasidone, quetiapine, pimozide, fluphenazine, haloperidol, ecopipam, sulpiride, and tiapride), VMAT-2 inhibitors (including tetrabenazine, deutetrabenazine, and valbenazine), botulinum toxins, and potentially the cannabinoids are considered tier 2 medications and reserved for more difficult to control symptoms (3). The use of pharmacotherapy can be limited by side effects such as sedation, drug-induced extrapyramidal symptoms, weight gain, and metabolic problems, leading to obstacles and even cessation. Thus, prescribing medication should be thoroughly considered whether tics causing interference on physical, social, or emotional interference determine the QoL.

So far, studies regarding the efficacy of delivering medication treatment via telemedicine to TS patients are lacking. Nevertheless, since TS and psychiatric disorders overlap in medication management, especially ADHD, we can look at the efficacy of tele-pharmacotherapy in the realm of psychiatry, which is meant to be the most frequently requested service (9, 92). Myers's study brings concrete evidence on the adherence to guideline-based care to children with ADHD and demonstrates high efficacy of providing pharmacotherapy remotely (49, 93, 94). Moreover, the ability to deliver medication management via videoconferencing has been proven in variable psychological conditions, such as depression (78, 95–97), PTSD (98), and other psychiatric conditions (45, 99). There is growing acceptance that telemedicine has successfully implemented medication management in patients with psychiatric disorders (100). Thus, there are reasons to believe that telemedicine can allow TS specialists to provide guideline-based medication treatment. However, potential challenges should be addressed, such as establishing a physician–patient relationship, coordinating with the complement mental health care system, etc. (100).

### Tele-Programming DBS

In recent years, DBS has been considered a last resort treatment option for severe TS resistant to medical and behavioral therapy (2). There is currently no consensus on the optimal brain target for tics treatment; however, our studies indicated that targeting the anteromedial globus pallidus (am GPi) or thalamic centromedian-parafascicular (CM-Pf) are more likely to reduce tic severity (101). Although utilizing DBS in TS management is still investigational, DBS has reached an innovative era. A recent study shows the reliability of predicting surgical candidacy (dystonia, essential tremor, and Parkinson's disease) for DBS through telemedicine technology and saved travel and time (102). To relieve the burden of postoperative follow-up from both sides of patients and physicians (103), engineers and clinicians have established a wireless system for DBS (104, 105), enabling DBS programmers to adjust parameters of the stimulators remotely. Studies from China (106) and Canada (107) confirm the safety and feasibility of using telemedicine for DBS follow-up. According to a recent report from China, among a total of 2,126 telemedicine visits since December 2019, 1,256 teleprogramming visits have performed, mainly for Parkinson's disease, saving a mean travel distance of 1,141 km (105).

## Limitations

Although the benefits of offering CBIT/HRT via telemedicine for individuals with TS/tics have been established (25, 88, 89), long-term prospective RCTs are required to study tele-CBIT/HRT and to compare this to traditional face-to-face care. Moreover, scientific evidence demonstrating the feasibility, efficacy, satisfaction, and economic value of telemedicine for TS is lacking significantly. Studies should assess the feasibility of delivering TS visits via telemedicine, which will be measured by the proportion of patients completing the assigned number of visits via telemedicine vs. in person. Also, clinicians will analyze the percentage of visits completed as scheduled, the proportion of visits completed via telemedicine vs. face-to-face office visits. Research and data are desired to measure economic benefits, such as time expenditure for each study visit, travel distance, and caregiver burden. Studies should cover more aspects of tele-treatment, including the efficacy of psychoeducation and medication management, therapeutic alliance, and adherence.

Additionally, the technology issue is still the primary concern to deliver health care for TS patients via telemedicine, regardless of the rapid advancement of technology, due to the uneven distribution of broadband. This barrier has been mentioned in multiple articles (88, 108, 109). TS patients present with sudden, rapid, non-rhythmic movements (motor tics) and vocalizations (phonic tics) that wax and wane in frequency. Thus, high-quality video and audio communication are essential in the interactions between health care providers and patients through telemedicine to precisely detect physical examination findings and perform adequate psychoeducation and behavior therapy without any disruption and delaying. According to the American Telemedicine Association (ATA) (110), telemedicine service should provide at a bandwidth of a minimum of 384 kbps in each of the downlink and uplink directions. However, the 2016 broadband progress report (111) from US Federal Communications Commission still showed a dramatic disproportion in bandwidth speed and internet access in rural/urban areas, which may impede providing adequate care for patients living far away from metropolitan areas through telemedicine. 5G, the 5th generation of the wireless network, will enable high-speed virtual connection with low latency. Ideally, a wearable device could be developed to identify quantified vocal and motor tics and would be an excellent asset for TS telemedicine.

Reimbursement and licensure have always been considered a significant barrier and were evolving in recent years. In the USA, states can reimburse Medicaid covered telemedicine services (112). Notably, most TS patients are not covered by Medicaid and have to be responsible for telemedicine costs by their families. Still, the reimbursement varies from states about what telemedicine types to include, whether and where they can be covered, etc. Thus, cost-effectiveness should be specifically assessed to demonstrate whether telemedicine can save money compared to traditional visits. Regarding licensure, generally, telemedicine is prohibited from practicing out-of-states. The Interstate Medical Licensure Compact is launched to streamline the licensing process and allow physicians to practice in multiple states, supporting the use of telemedicine. Currently, 29 states, Washington D.C., and the Territory of Guam are involved in the Compact (113). Other common concerns mentioned in previous studies include privacy/safety and establishing rapport, which should carefully be addressed in future studies in TS (114).

## Conclusion

**Figure 1:**
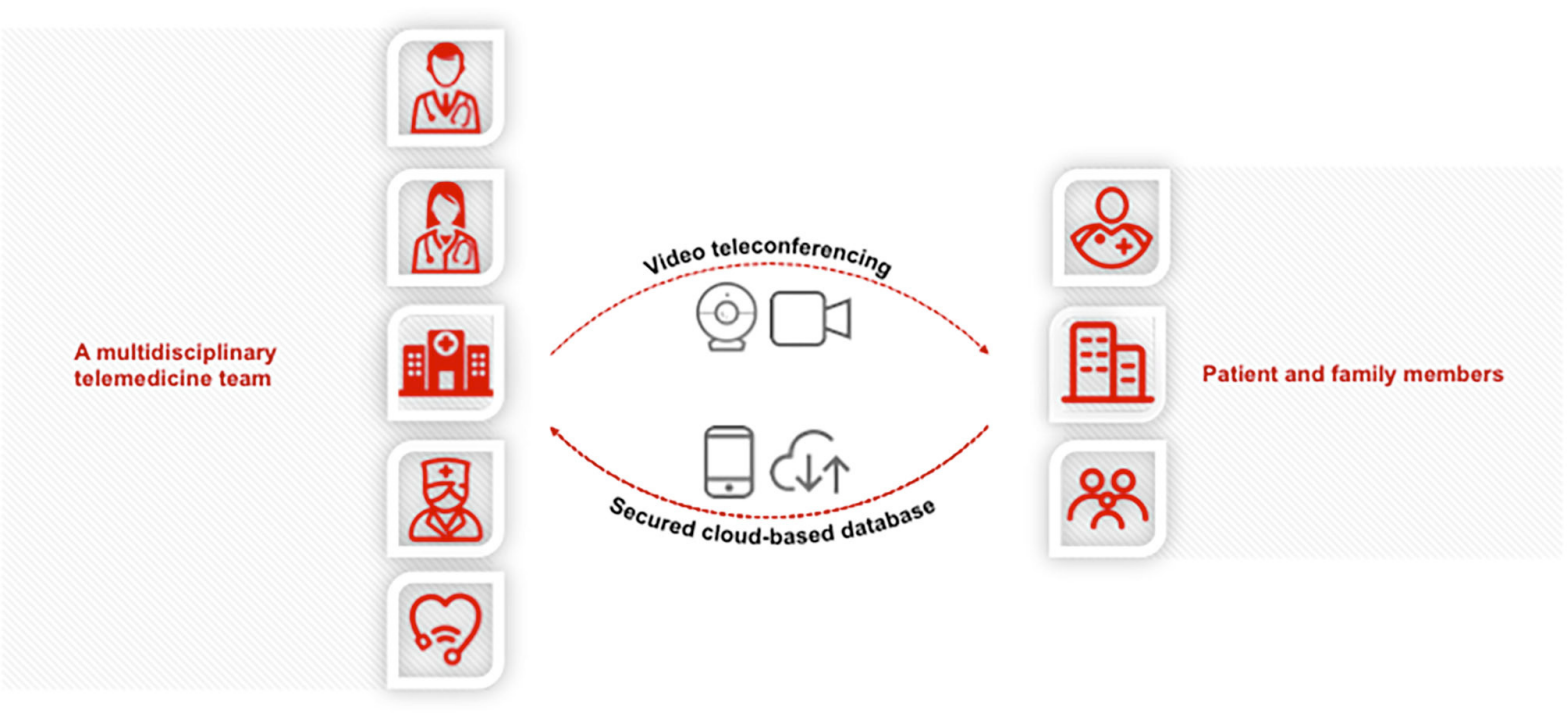
Schema of multidisciplinary care for the Tourette syndrome (TS) via telemedicine. Video telemedicine can be used to provide comprehensive care and support for TS patients and their families/caretakers in a timely and easily accessible fashion while ensuring patient privacy and HIPAA compliance. Neurologists and psychiatrists can evaluate the patients and offer management plans and education remotely. Behavioral therapists and psychologists can provide the Comprehensive Behavioral Intervention for Tics (CBIT), other psychosocial and behavioral interventions, and psychoeducation via telemedicine. Additional specialty service and social work needs can also be accommodated via telemedicine as appropriate. In summary, telemedicine promises to be a revolutionary approach to improve access to care for patients with TS, especially those living in more rural areas. Its feasibility, satisfaction, clinical effects, and cost-effectiveness should be assessed by further research. Potential limitations such as technological issues, reimbursement, and licensure should be addressed as well.

So far, telemedicine has been studied in the realm of movement disorders, mainly evaluated in PD, and proved to be a feasible alternation to in-person visits. As PD, TS is most likely valid for evaluation and management via video conferencing, yet telemedicine has been sparsely studied in this field. Tele-CBIT holds promise, though further prospective RCTs are required to conclude. Telemedicine has potential prospects in contributing to multidisciplinary care for TS patients who face physical or economic access issues. As in Figure 1, we propose a multidisciplinary telemedicine team for the care of patients with TS, including neurologists, psychiatrists, psychologists, and behavioral therapists. They can evaluate patients, offer management plans, provide psychosocial and behavioral interventions such as CBIT, and offer education to the patients' peers, family members, and teachers remotely via videoconferencing technology.

## Author Contributions

All authors listed have made a substantial, direct and intellectual contribution to the work, and approved it for publication.

## Conflict of Interest

The authors declare that the research was conducted in the absence of any commercial or financial relationships that could be construed as a potential conflict of interest.
